# Differential effects of PCSK9 loss of function variants on serum lipid and PCSK9 levels in Caucasian and African Canadian populations

**DOI:** 10.1186/1476-511X-12-70

**Published:** 2013-05-10

**Authors:** Janice Mayne, Teik Chye Ooi, Angela Raymond, Marion Cousins, Lise Bernier, Thilina Dewpura, Francine Sirois, Majambu Mbikay, Jean Davignon, Michel Chrétien

**Affiliations:** 1 Ottawa Institute of Systems Biology, Department of Biochemistry, Microbiology and Immunology, University of Ottawa, 451 Smyth Road, Ottawa, ON K1H 8M5, Canada; 2 Clinical Research Laboratory, Division of Endocrinology and Metabolism, Department of Medicine, The Ottawa Hospital, University of Ottawa, 1967 Riverside Drive, Ottawa, ON K1H 7W9, Canada; 3 Chronic Disease Program, Ottawa Health Research Institute, The Ottawa Hospital, University of Ottawa, Ottawa, ON, Canada; 4 Hyperlipidemia and Atherosclerosis Research Group, Clinical Research Institute of Montreal, Montreal, Quebec, Canada

**Keywords:** Compound heterozygotes, Hypercholesterolemia, Hypocholesterolemia, Low density lipoprotein receptor, PCSK9, Single nucleotide polymorphisms

## Abstract

**Objectives:**

Variants of the secreted glycoprotein, proprotein convertase subtilisin/kexin 9 (PCSK9), associate with both hypo- and hyper-cholesterolemic phenotypes. Herein, we carried out full exonic sequencing of *PCSK9* documenting the frequency of single and multiple *PCSK9* variations and their effects on serum lipoprotein and PCSK9 levels in Caucasian Canadians.

**Methods:**

The 12 exons of *PCSK9* were sequenced in 207 unrelated Caucasian Canadians. Minor allele frequencies of *PCSK9* variants were compared amongst LDL cholesterol (LDLC) quintiles. Serum PCSK9 levels were measured by ELISA and lipoproteins by enzymatic methods. Comparisons were made with a Caucasian family cohort (n = 51) and first generation African Canadians (n = 31).

**Results:**

In Caucasians, but not African Canadians, the c.61_63insCTG (denoted L10Ins) and A53V PCSK9 variations were linked and their frequency was significantly higher among Caucasian Canadians with LDLC levels in the <25th percentile. In both the unrelated and family Caucasian cohorts those carrying the L10A53V PCSK9 variant had significantly lower LDLC without reduction in plasma PCSK9. The I474V PCSK9 variant associated with significantly lower serum PCSK9 and LDLC. A novel PCSK9 variant was identified; E206K. We found that the frequency of multiple PCSK9 variations was higher in first generation African Canadians.

**Conclusions:**

We showed that the L10A53V and I474V PCSK9 variants were significantly associated with lower LDLC levels in Caucasian Canadians but differed in their effect on serum PCSK9 concentrations, illuminating differences in their mechanism of inaction and indicating that that PCSK9 measurement alone may not always be a good indicator of PCSK9 function.

Full exonic sequencing of *PCSK9* pointed to factors that may contribute to L10Ins PCSK9 variant loss of function in Canadians of Caucasian but not those of African descent. These included; (1) its tight linkage with the A53V variant in Caucasians and/or (2) for both the L10 and I474V, the combined (and negating) effect of multiple, differing phenotypic PCSK9 variants within individuals of African ancestry for which combinations of PCSK9 variations and their overall frequency was higher. No population studies, to our knowledge, have addressed or accessed the effect of multiple PCSK9 variants on cholesterol profiles. Our results indicate that this should be considered.

## Background

Elevated low density lipoprotein cholesterol (LDLC) levels are recognized as a major risk factor for coronary artery disease [1,2]. Cell surface liver low density lipoprotein receptors (LDLR) clear LDL particles from circulation by binding LDL particles through its protein component, apolipoprotein B100 (ApoB100), followed by endocytosis [3]. LDL is released from the LDLR in the endosome and travels to the lysosome for degradation, while the LDLR recycles to the cell surface. Gene variations in *LDLR* (*low density lipoprotein receptor*) and *APOB* (*apolipoprotein B100*) were identified as causes of autosomal dominant hypercholesterolemia (ADH) and named familial hypercholesterolemia (FH) 1 and FH2, respectively [2]. They account for approximately 80% of all FH cases [4,5]. The FH3 locus was identified in 2003 by Abifadel *et al.* as *PCSK9* (*Proprotein Convertase Subtilisin*/*kexin type 9*) [6].

PCSK9 is the ninth member of a family of endoproteolytic enzymes important in development and normal physiology due to their regulated proteolytic maturation of proproteins including neuropeptides, hormones, cytokines, growth factors, receptors, cell surface and serum proteins [7,8]. The 12 exons of *PCSK9* encode a 692 amino acid secreted glycoprotein [9]. PCSK9, like its family members, is synthesized as a preproprotein containing several well-defined motifs including a pre-signal, pro-inhibitory, catalytic and c-terminal domain [10]. Following autocatalytic cleavage, PCSK9 and its inhibitory pro domain are secreted as a heterodimer [9]. Therefore, unlike its family members, PCSK9 does not function as an enzyme and instead plays a ‘moonlighting’ role as an escort protein [11,12]. PCSK9 binds to LDLR at the cell surface [13] and escorts it from the endosomal recycling pathway toward the lysosomal compartment for degradation, thereby modifying circulating LDLC levels [14-17]. Regions within PCSK9’s catalytic, pro and c-terminal domains have been implicated in the equilibrium between PCSK9:LDLR binding at the cell surface and in the endosome [13,18-21]. PCSK9 variants have been identified in each of these domains, some of which associate with significant changes in circulating LDLC levels; PCSK9 variants that associate with hypercholesterolemia or ADH [22-26] are termed ‘gain of function’, while PCSK9 ‘loss of function’ variants associate with hypocholesterolemia and reduced risk of coronary artery disease [27-33].

Minor allele frequencies (MAF) of ‘gain of function’ PCSK9 variants that associate with ADH are relatively low and account for approximately 2.3% of ADH [4,34]. ‘Loss of function’ PCSK9 variations that result in very low cholesterol levels (ie <5th percentile) like the C679X and Y142X found in African Americans occur with a combined MAF of 2% in that population [28]. Phenotypically milder ‘loss’ and ‘gain of function’ PCSK9 variants are more widespread, and their MAFs and phenotypic influence can vary with ethnicity [28,31,32,35,36]. For instance, the PCSK9 R46L variant associates with lower LDLC levels in Caucasian populations at a MAF of 1.6% but at 0.28% in African Americans [30]. The PCSK9 A443T that associates with lower LDLC levels in African Americans (MAF 9.4%) is found at 0.048% in Caucasians. The PCSK9 c.61_63insCTG variant (denoted L10Ins) is found at similar MAFs (between 11 and 15%) in Caucasians [32] and Japanese [31], but associates with lower LDLC levels in Caucasians [32].

We recently observed several common loss of function PCSK9 variants in a French Canadian cohort (including L10insA53V, R46L and I474V) in addition to a novel, strong loss of function PCSK9 Q152H variant [37,38]. This variant precludes proPCSK9 processing and secretion and has a dominant negative effect on wildtype PCSK9 [37]. In this paper we extend these findings by determining which *PCSK9* variations associated with low LDLC levels (<25th percentile) in a mixed Caucasian Canadian population and compared their frequency in Caucasian Canadians with LDLC in the 25-49th, 50-74th, 75-95th and >95th percentiles (age and gender matched). We investigated the influence of *PCSK9* variants on serum lipid and PCSK9 levels in this population and in a large Caucasian family. To begin to study ancestry and *PCSK9* variations, we compared our above results with *PCSK9* variations found in first generation African Canadians. Unlike many studies on the influence of PCSK9 variants on serum lipid levels, genotyping of our subjects is based on complete exonic sequencing of the *PCSK9* gene and not selective sequencing for a particular variation. This is an important consideration when studying a polymorphic gene like *PCSK9*.

## Results

### Clinical characteristics of low density lipoprotein cholesterol (LDLC) quintiles in an unrelated Caucasian Canadian population

**Figure 1:**
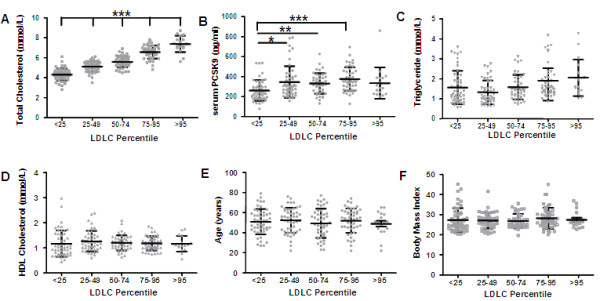
**Scatter plot representation of mean ± SD of serum lipid and PCSK9 concentrations as well as age and BMI in a Caucasian Canadian population by LDL Cholesterol quartile, adjusted for age and gender.** Comparisons of the <25th LDLC percentile with other LDLC subgroups by Kruskal-Wallis Test with Dunn’s Multiple Comparison Post Test. Significance is indicated as *p < 0.05, **p < 0.001, ***p < 0.0001.

Figure 1 shows scatter plot representation of the mean ± SD for lipids, serum PCSK9, body mass index (BMI) and age of LDLC sub-groups; <25th (n = 51), 25-49th (n = 48), 50-74th (n = 45), 75-95th (n = 46) and >95th (n = 17) percentiles, adjusted for age and gender. The <25th LDLC percentile group was compared to other sub-groups and statistical differences noted (Figure 1). As expected TC (Panel A) was significantly increased in each LDLC sub-group in comparison to the <25th LDLC percentile sub-group. Panel B shows that plasma PCSK9 in the <25th LDLC percentile sub-group (260.8 ± 103.5 ng/ml) was significantly lower than the 25-49th (345.2 ± 158 ng/ml, p < 0.05), 50-74th (330.7 ± 102 ng/ml, p < 0.001) and 75-95th (375.1 ± 117.6 ng/ml, p < 0.0001) LDLC percentile sub-groups. However, although lower, plasma PCSK9 in the <25th LDLC percentile sub-group (260.8 ng/ml ± 103.5) was not significantly different from the >95th LDLC percentile subgroup (Panel B; at 334.4 ng/ml ± 156). Triglyceride and HDLC levels did not differ between LDLC sub-groups, nor did BMI and age (Panels C-F, respectively). Serum PCSK9 was positively correlated with TC (*r* = 0.3919, p < 0.0001), LDLC (*r* = 0.3312, p < 0.0001) and HDLC (*r* = 0.2647, p = 0.0001) but not TG, age and BMI (Additional file 1: Figure S1). Our sample population is largely Caucasian based on self-reporting but would be multiethnic in ancestry; 25% British, 22% French, 24% Irish, 20% other Europeans, 7% Asian and 2% other (Statistics Canada).

### PCSK9 variations in the Caucasian Canadian population

Table 1 shows the PCSK9 exonic variants identified in our Caucasian Canadians and their percent MAF (%) sub-divided by LDLC quintiles, and in the whole population. We identified known PCSK9 variants including the R46L and L10Ins associated with reduced LDLC in Caucasians [32] and the I474V and E670G not reported to affect LDLC in Caucasians. Our MAF (%) for these variations are similar to those previously reported; 1.9 for R46L versus 1.6 in [30]; 15.5 for L10Ins versus 12.8 in [32]; 12.6 for I474V versus 18 in [30]; and 3.6 for E670G versus 3.4 in [39]. In our Caucasian Canadians -and as reported for European and Japanese populations- the L10ins [within the signal peptide domain of PCSK9] showed linkage disequilibrium with the A53V variation in the prodomain of PCSK9 [31,32]. We identified a PCSK9 variant R93C reported in a Japanese population to associate with low LDLC [31]. This individual reported distant Asian ancestry. We found the H553R PCSK9 variant that associates with increased LDLC [30] in an individual in >95th percentile LDLC sub-group and of Latin American ancestry. One individual carried a novel PCSK9 variant E206K (95th percentile for LDLC). Table 2 shows the frequency of single and multiple PCSK9 exonic variants in the Caucasian cohort. 101 of our 207 Caucasian participants (48.7%) did not carry any exonic PCSK9 variant. 71 or 34.3% carried one PCSK9 variant. 35 of our 207 Caucasian participants (16.9%) carried two or more PCSK9 exonic variants. The effect of PCSK9 variants on lipoprotein parameters has been reported in several populations, but to our knowledge no study has addressed how the presence of multiple PCSK9 variants affects our understanding of the loss- and gain- of function of PCSK9 variation in larger population studies. In our population we cannot say whether these multiple variations occur on the same or separate alleles.

**Table 1:** Comparison of minor allele frequencies (%) for PCSK9 exonic single nucleotide variations in a Caucasian Canadian population subdivided by LDLC quintiles

**LDLC percentile group**	**Group < 25th**	**25-49th**	**50-74th**	**75-95th**	**>95th**	**All**
	**(n = 51)**	**(n = 48)**	**(n = 45)**	**(n = 46)**	**(n = 17)**	**(n = 207)**
**PCSK9 Variant**	**Sub-Group Minor Allele Frequencies (MAF %)**					**All MAF (%)**
**L10Ins/A53V**	**20.6**	10.4*	10.0*	7.6**	2.9*	**15.5**
**L10Ins/A53V (Homo)**	NF	NF	2.2	1.1	5.9	1.9
**L10Ins**	NF	1.0	NF	1.1	NF	0.24
**R46L**	2.9	1.0	3.3	1.1	NF	0.24
**R93C**	0.98	NF	NF	NF	NF	0.24
**E206K**	NF	NF	NF	1.1	NF	0.24
**I474V**	15.7	12.5	16.7	7.6	5.9	12.6
**I474V(Homo)**	2.0	NF	3.3	NF	NF	2.4
**H553R**	NF	NF	NF	NF	5.9	0.24
**E670G**	2.9	4.2	4.4	2.2	5.9	3.6

*MAF*, minor allele frequency; *NF*, not found; Fisher’s Exact Test comparing <25thpercentile MAF to each other group *p = 0.033 (25-49th); *p = 0.046 (50-74th); **p = 0.0067 (75-95th); *p = 0.037 (>95th); Chi-squared 0.016 comparing all groups.

**Table 2:** Frequency of single and multiple PCSK9 exonic variations in Caucasian and African Canadian populations

**PCSK9 Variant**	**PCSK9 Variant**
**Combined frequency (%) in Canadian population**	**Combined frequency (%) in African population**
**No PCSK9 Exonic Variations (n = 101) 48.7%**	**No PCSK9 Exonic Variations (n = 6) 19.4%**
**Carriers of Single PCSK9 Exonic Variations (n = 71) 34.3%**	**Carriers of single PCSK9 ExonicVariations (n = 8)** ** 25.8%**
**L10Ins/A53V (n = 26)**	**L10Ins (n = 3)**
**L10Ins (n = 1)**	**A443T (n = 1)**
**R46L (n = 1)**	**I474V (n = 2)**
**I474V (n = 33)**	**H417Q (n = 1)**
**R93C (n = 1)**	**H553R (n = 1)**
**E670G (n = 9)**	**E670G (n = 1)**
**Carriers of Multiple PCSK9 Exonic Variations (n = 35) 16.9%**	**Carriers of Multiple PCSK9 ExonicVariations (n = 17) 54.8%**
**L10Ins/A53V -L10Ins/A53V (n = 3)**	**L10Ins/R469W (n = 1)**
**L10Ins/A53V -L10Ins/A53V –I474V (n = 1)**	**L10Ins/I474V/H553R (n = 1)**
**L10Ins/A53V -I474V (n = 13)**	**L10Ins/I474V/E670G (n = 2)**
**L10Ins/A53V -E206K -I474V (n = 1)**	**L10Ins/N425S/I474V (n = 2)**
**L10Ins/A53V -I474V -I474V (n = 1)**	**L10Ins/R469W/E670G (n = 1)**
**L10Ins/A53V -R46L (n = 4)**	**L10Ins/E670G(Homo) (n = 1)**
**L10Ins/A53V -R46L -I474V (n = 2)**	**L10Ins(Homo)/I474V (n = 1)**
**L10Ins/A53V -E670G (n = 1)**	**L10Ins(Homo)/E670G (n = 1)**
**L10-E670G (n = 1)**	**L10Ins/A443T/I474V/E670G (n = 1)**
**R46L -I474V (n = 1)**	**L10Ins/A443T(Homo)/H553R/Q619P (n = 1)**
**R46L -E670G (n = 1)**	**L10Ins(Homo)/I474V/E670G(Homo) (n = 1)**
**I474V -I474V (n = 4)**	**A443T/I474V (n = 1)**
**I474V -E670G (n = 2)**	**A443T/C679X (n = 1)**
	**I474V/E670G (n = 1)**
	**E670G(Homo) (n = 1)**

### PCSK9 variations in the African Canadian population

We compared the findings in our Caucasian group to 31 individuals who self-identified as first generation African Canadians. Table 3 shows the PCSK9 variants identified, their MAFs and previously reported effect on LDLC levels in those of African ancestry. One carried the H417Q and two the R469W PCSK9 variations associated with increased LDLC levels in African Americans [30]. Four carried the A443T PCSK9 variant and one of these the C679X variant associated with reduced LDLC in African Americans [28,30]. The E670G and I474V are common polymorphisms in this population with MAFs of 21 and 19.4%, respectively. This is similar to their reported frequency in African Americans [30] and, for the E670G, this variant is significantly more frequent in our African than Caucasian Canadian cohorts (p < 0.0001). The L10Ins was a common variant in our African Canadian population - 13 individuals were heterozygous and 3 were homozygous for the L10Ins. The combined MAF in this group for the L10Ins is 30.7%. Notably, none carried the A53V PCSK9 variant (and −64 C > T variation from ATG) that is linked in the Caucasian population. The frequency of heterozygosity and homozygosity for the L10Ins in the African Canadian population (41.9% and 9.7%, respectively) was higher than the Caucasian Canadian population (22.7% and 1.9%, respectively; p = 0.0044). The frequency of the I474V variant did not vary between our African and Caucasian cohorts.

**Table 3:** Minor allele frequencies (%) of PCSK9 exonic variations found in African Canadians

**PCSK9 Variant**	**MAF n = 31**	**Reported MAF (%) in African Americans**^**b**^	**Reported effect on LDLC levels in African Americans**^**b**^
**L10Ins**^**a**^	21 or 30.7 combined		ns
**L10Ins (Homozygous)**	9.7		
**H417Q**	1.6	0.3^b^	↑ 10%
**A443T**	6.5 or 9.7 combined	9.4^b^ combined	↓ 4%
**A443T (Homozygous)**	3.2		↓ 30%
**N425S**	3.2	1.9	nd
**I474V**	19.4	22^b^	ns
**R469W**	3.2	0.75^b^	nd
**H553R**	4.8	1.3	↑ 10%
**E670G**	11.3 or 21.0 combined	26^b^ combined	↑ 4%
**E670G (Homozygous)**	9.7		↑ 30%
**C679X**	1.6	0.72^b^	↓ 40%

^a^The L10Ins was NOT linked to the A53V variation and –64C/T intronicvariation from ATG as seen in Canadian Caucasian Population. ^b^*MAF*, minor allele frequency reported by Kotowskiet al 2006. *ns*: not significant; *nd*: not determined.

Table 2 shows the frequency of single and multiple PCSK9 exonic variants in our African Canadian population. Six of 31 individuals, or 19.4%, did not carry exonic variations in *PCSK9*, while 25.8% carried a single *PCSK9* variation and a striking 54.8% carried multiple *PCSK9* variations. Statistically significant differences were observed in the African and Caucasian Canadian populations when comparing the frequency of multiple PCSK9 variants (p < 0.0001).

### Influence of PCSK9 variations on lipoproteins in the Caucasian Canadian population

When compared to the other LDLC percentile groups, the frequency of the L10InsA53V variant was significantly associated with the <25th percentile (Table 1: Fisher’s Exact Test comparing <25th percentile MAF to each other group *p = 0.033 (49-25th); *p = 0.046 (74-50th); **p = 0.0067 (95-75th); *p = 0.037(>95th); Chi-squared *p = 0.016 comparing all groups). The frequency of PCSK9 variants R46L, I474V and E670G were not associated with any LDLC sub-group (Table 1). The one individual who carried the R93C variant was in the <25th LDLC percentile, a variant associated with low LDLC levels in a Japanese population [31]. The novel variant E206K was identified in a single individual with LDLC at the 95th percentile. On-going family and cell biology studies will conclude whether it exerts a gain of function in PCSK9.

The occurrence of single and multiple PCSK9 variants in the Caucasian population (Table 2) prompted us to analyze the effect of our most common PCSK9 variants (termed ‘carriers’ but not exclusively of that variant) on lipoprotein parameters in comparison to those that do not carry that PCSK9 variation (but may carry others and termed ‘non- carriers’ of said variation (Table 4). Then to exclude the influence of multiple PCSK9 variants on a particular PCSK9 variant and its effect on lipoprotein parameters, we carried out the more vigorous comparison of individuals who carry a single variation in *PCSK9* (termed ‘X’ variant) with those who carry no variation in *PCSK9* (termed no variant; Table 5). In Table 4, only the L10A53V PCSK9 carriers show significantly lower TC (9.5%; p = 0.0047) and LDLC (16.1%; p < 0.0002) concentrations when compared to non-carriers. However, the more vigorous comparison of only a single PCSK9 variant versus no variant (Table 5) shows not only that the L10A53V PCSK9 carriers show significantly lower TC (12.5%; p = 0.0032) and LDLC (19.8%; p = 0.0005) but those that carry exclusively the I474V have significantly lower LDLC (9.9%; p = 0.05) and those carrying only the E670G variant have significantly lower TC (16.2%; p = 0.043) and TG (27.7%; p = 0.039) levels.

**Table 4:** Comparison of serum lipid and PCSK9 concentrations in non-carriers versus carriers of a given PCSK9 variant values

**PCSK9 Genotype**	**Total Cholesterol (mmol/L)**	**p-value**	**Triglyceride (mmol/L)**	**p-value**	**HDL Cholesterol (mmol/L)**	**p-value**	**LDL Cholesterol (mmol/L)**	**p-value**	**Serum PCSK9 (ng/mL)**	**p-value**
**Non-Carrier** (n = 155)	**5.67 ± 1.16**		1.54 ± 0.73		1.19 ± 0.35		**3.78 ± 1.00**		327.2 ± 130.6	
**L10A53V**		***0.0047***		*0.56*		*0.63*		***0.0002***		*0.76*
**Carrier** (n = 52)	**5.13 ± 0.99**		1.66 ± 0.82		1.21 ± 0.49		**3.17 ± 0.92**		326.5 ± 133.0	
**Non-Carrier** (n = 150)	5.59 ± 1.19		1.54 ± 0.75		1.19 ± 0.37		3.70 ± 1.06		**338.6 ± 136.2**	
**I474V**		*0.13*		*0.57*		*0.86*		*0.056*		***0.033***
**Carrier** (n = 57)	5.35 ± 0.98		1.61 ± 0.76		1.21 ± 0.44		3.42 ± 0.86		**296.7 ± 111.0**	
**Non-Carrier** (n = 192)	5.56 ± 1.14		1.59 ± 0.77		1.20 ± 0.40		**3.64 ± 1.01**		328.1 ± 127.0	
**E670G**		*0.19*		*0.13*		*0.36*		*0.59*		*0.44*
**Non-Carrier** (n = 15)	5.11 ± 1.12		1.27 ± 0.48		1.10 ± 0.30		3.45 ± 1.11		313.8 ± 177.9	

Variant values represent mean ± SD. Differences between groups were determined by Mann Whitney test with level of significance as p < 0.05.

**Table 5:** Comparison of serum lipid and PCSK9 concentrations in non-carriers of any PCSK9 variant versus carriers of only a given PCSK9 variant values

**PCSK9 Genotype**	**Total Cholesterol (mmol/L)**	**p-value**	**Triglyceride (mmol/L)**	**p-value**	**HDL Cholesterol (mmol/L)**	**p-value**	**LDL Cholesterol (mmol/L)**	**p-value**	**Serum PCSK9 (ng/mL)**	**p-value**
**No Variant** (n = 101)	**5.82 ± 1.15**		1.55 ± 0.72		1.19 ± 0.34		**3.93 ± 0.97**		341.0 ± 128.9	
**L10A53V**		***0.0032***		*0.82*		*0.64*		***0.0005***		*0.84*
**Only** (n = 29)	**5.09 ± 0.96**		1.67 ± 0.86		1.18 ± 0.41		**3.15 ± 0.96**		352.7 ± 138.5	
**No Variant** (n = 101)	5.82 ± 1.15		1.55 ± 0.72		1.19 ± 0.34		**3.93 ± 0.97**		341.0 ± 128.9	
**I474V**		*0.097*		*0.53*		*0.86*		***0.050***		*0.087*
**Only** (n = 37)	5.48 ± 1.01		1.67 ± 0.81		1.18 ± 0.39		**3.54 ± 0.90**		301.3 ± 110.2	
**No Variant** (n = 101)	**5.82 ± 1.15**		**1.55 ± 0.72**		1.19 ± 0.34		3.93 ± 0.97		341.0 ± 128.9	
**E670G**		***0.043***		***0.039***		*0.69*		*0.14*		*0.34*
**Only** (n = 9)	**4.88 ± 1.01**		**1.12 ± 0.41**		1.14 ± 0.37		3.24 ± 1.08		329.3 ± 227.8	

Variant values represent mean ± SD. Differences between groups were determined by Mann Whitney test with level of significance as p < 0.05.

### Influence of PCSK9 variations on serum PCSK9

To date, the effect of most PCSK9 variants on serum PCSK9 concentrations has not been reported. In 2009, Humphries and colleagues reported that the R46L and D374Y PCSK9 variants both exhibit lower serum PCSK9 concentrations despite having opposite effects on LDLC levels [40]. Herein, we examined the influence of frequent PCSK9 variants found in our Canadian Caucasian population on serum PCSK9 levels. Table 4 shows that although the presence of the L10InsA53V variant does not affect circulating PCSK9 levels (326.5 ± 133.0 (carrier) vs 327.2 ± 130.6 ng/ml (non-carrier), carriers do have significantly lower LDLC concentrations (16.1%; p = 0.0002). This significance remained when comparing individuals carrying only the L10InsA53V variant versus those with no *PCSK9* variation. In this instance, serum PCSK9 again remained unchanged (p = 0.84) while LDLC were significantly lower by 19.8% (p = 0.0005; Table 5). Table 5 shows that while the presence of the I474V variation lowered serum PCSK9 levels by 11.6%, but not significantly so (p = 0.087), these carriers have significantly lower LDLC levels (9.9%; 3.54 ± 0.90 (I474V only) vs 3.93 ± 0.97 mmol/l (no variant) p = 0.050). Interestingly, carriers with only the E670G variant showed a non-significant difference in serum PCSK9 but significantly lower TG concentrations (27.7%; p = 0.039) not seen for either the L10A53V nor I474V carriers (Table 5).

### Influence of PCSK9 variations on lipoprotein parameters and plasma PCSK9 in a Caucasian family of French ancestry

**Figure 2:**
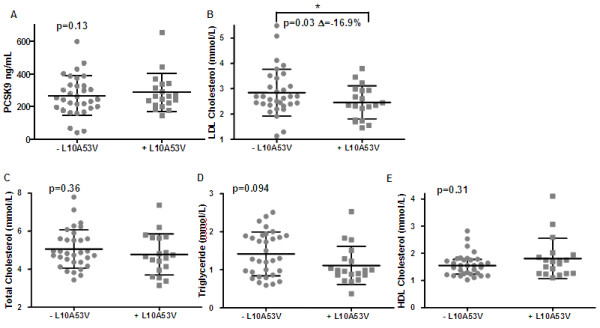
**Scatter plot representation of mean ± SD of serum lipid and PCSK9 concentrations as well as age and BMI in a French Canadian pedigree.** –L10A53V and +L10A53V represents non-carriers and carriers of that PCSK9 variant, respectively. Panel **A**; shows the levels of plasma PCSK9 and panels B-E lipids (**B**; LDL cholesterol, **C**; Total Cholesterol, **D**; Triglyceride and **E**; HDL cholesterol) for these two groups. Differences between groups were determined by Mann Whitney test with level of significance as p < 0.05.

To assess whether the results seen in the general Caucasian population also applied to a family, we measured the lipoproteins, plasma PCSK9 and sequenced the 12 exons of *PCSK9* in a French Caucasian pedigree of 51. Nineteen of the 51 participants (37.3%) carried the L10A53V PCSK9 variant. Three carried the phenotypic strong loss of function Q152H PCSK9 variant that we characterized in Mayne *et al.* (2011), which was associated with 79% lower plasma PCSK9 and 48% lower LDLC. The comparisons of plasma lipoproteins and PCSK9 of L10A53V carriers (+L10A53V) in comparison to non-carriers (−L10A53V) are shown in Figure 2. Similar to comparisons of the L10A53V variants in the unrelated Caucasian population, within this pedigree those that carried the L10A53V variant had significantly lower LDLC than those that did not carry this PCSK9 variant (16.9%, *p* = 0.03: 2.46 ± 0.15 vs 2.96 ± 0.16 ng/ml, respectively). No other lipoprotein parameter, nor age or BMI differed by L10A53V status in this pedigree. Again this reduction in LDLC was not due to reduced plasma PCSK9; L10A53V carriers had 286.0 ± 26.6 ng/ml and non-carriers 289.1 ± 19.4 ng/ml PCSK9.

## Discussion

There are several major findings from this study that compared *PCSK9* variations in Caucasian with first generation African Canadians. The first finding is co-segregation of the signal peptide c.61_63insCTG variation (L10Ins) with the prodomain A53V PCSK9 variation in Caucasian but not African Canadians. Other groups have reported the tight linkage of the L10Ins and A53V PCSK9 variations as we have observed here [31,32]. In Yue *et al.* (2006), the PCSK9 L10Ins variant also associated with lower LDLC levels [32]. An intronic -64C/T from ATG also co-segregated with the L10InsA53V variants in our Caucasian population and as seen previously in other populations [31,32]. This intronic PCSK9 variation was not seen in our African cohort. Hence, we report that the combination of these variations, the intronic -64C/T from ATG, the L10Ins and A53V are unique to our Caucasian and not African Canadian populations.

The second finding was that the frequency of the L10InsA53V variant in our Canadian population was significantly higher in our low LDLC group (<25th percentile) versus those with LDLC levels >25th percentile, age and gender matched (p = 0.016). This was not observed in African Canadians nor is this variation reported to affect LDLC levels in African Americans [30]. We propose that the tight linkage of the L10Ins with the prodomain A53V variant (and the intronic promoter -64C/T variation) may contribute to the reported association of the L10Ins with low LDLC in the Caucasian population but not in African Americans.

Thirdly, no study to our knowledge has reported the frequency of single and multiple *PCSK9* variation frequencies in their populations. This is an important consideration since the *PCSK9* gene is highly polymorphic with more than 50 exonic variations documented with opposing effects on LDLC levels [34]. Herein, we show that multiple *PCSK9* variations are present in both Caucasian and African populations but are significantly more frequent in Canadians of African descent. Whereas 48.7% of Caucasian Canadians did not carry any exonic variation in PCSK9, only 19.4% of our first generation African Canadians did not carry any exonic variation in PCSK9 (Table 2). 34.3% of our Caucasian population and 25.8% of our African population carried single *PCSK9* variations. Striking was the difference in the occurrence of multiple *PCSK9* exonic variations in our African population (54.8%) in comparison to 16.9% in Caucasians. This data prompted us to re-evaluate the effect of our most common PCSK9 variants in our Caucasian population (1) in the presence of other variants and (2) in the absence of multiple variants (Tables 4 and 5, respectively). In the presence of multiple PCSK9 variants only the L10InsA53V carriers showed significantly lower TC and LDLC levels (Table 2; 9.5%, p = 0.0047 and 16.1%, p = 0.0002, respectively). However, once we evaluated only those single variants against those that carried no exonic variant in PCSK9 (Table 5) those that carried the L10InsA53V variations showed a larger significant decrease of 19.8% LDLC (p = 0.0005) and those that carried the I474V variation a significant 9.9% decrease in LDLC (p = 0.050). Interestingly, when we compared the carriers of only E670G variants with those that carry no PCSK9 exonic variation, although LDLC levels were non-significantly decreased, their TG levels were 27.7% lower (p = 0.039). The positive correlation of plasma PCSK9 with TG has been reported in several population studies yet not others [41,42]. This difference may be attributable to the occurrence of the E670G, and indeed other variants in that population that may affect the degree of correlation to several lipoprotein parameters. For instance, in our Caucasian population, serum PCSK9 is positively correlated to LDLC (*r* = 0.3312, p < 0.0001); however when we measure the correlation of those carrying only the L10InsA53V variants, this correlation is lost (*r* = 0.2360, p = 0.2178).

This may not be surprising in light of our fourth finding that the measurement of plasma PCSK9 illuminates a difference in the mechanism of action of the L10A53V variation versus the I474V variation in lowering LDLC in our Caucasian population. We, and others, have shown that if plasma PCSK9 levels decrease it is often associated with decreased LDLC. This is true of the two African missense mutations, C679X and Y142X, the Caucasian Q152H variant and the R46L variant [28,37,40]. Circulating PCSK9 levels for carriers of the PCSK9 I474V variant were lower (11.6%) than the non-variant PCSK9 population, but not significantly so (p = 0.087). However, this decrease could contribute to the significantly lower LDLC in these individuals (9.9%, p = 0.050). In contrast, the L10A53V carriers did not exhibit any difference in circulating PCSK9 concentrations despite a 19.8% reduction in comparison to persons who do not carry any variation in PCSK9 (p = 0.0005). Again, this points to a different mechanism of inaction for the loss of function PCSK9 L10A53V variant.

## Conclusions

In conclusion, this study highlights differences in PCSK9 variants found and their frequencies among Caucasian and African Canadians, and is the first to report on the frequency of multiple PCSK9 variants. The frequency was significantly greater in African Canadians probably reflecting selective historical environmental pressures on this group. Given these findings further investigation to catalog specific combinations of PCSK9 variants in various populations and large scale studies will be required to evaluate the effect of multiple variants on PCSK9 biosynthesis, secretion and action toward the LDLR and their combined effect on circulating cholesterol levels and profiles, as opposed to studies of single variants.

## Materials and methods

### Population study participants

With informed written consent, blood samples and clinical measurements were obtained for subjects using Ottawa Hospital Research Institute (OHRI) and Clinical Research Institute of Montreal (IRCM) ethics committee approved study protocols. No participants were on any lipid-lowering medications. We obtained blood samples from 238 participants (128 men and 110 women; 207 self-reporting as Caucasian and 31 self-reporting as African Canadian) recruited by the Ottawa Hospital Lipid Clinic and 51 participants (25 men and 26 women; Caucasian family of French Ancestry) recruited from the IRCM following a 12 hr fast. 207 unrelated individuals self-reporting as Caucasian were grouped with LDLC levels in the <25th (n = 51), 25-49th (n = 48), 50-74th (n = 45), 75-95th (n = 46) and >95th (n = 17) LDLC percentile, age and gender matched, as provided in the Lipid Research Clinics Table [43]. Individuals underwent anthropometric measurements including height and weight. Body mass index (BMI) was calculated according to the formula: BMI = weight (kg)/height (m)^2^.

### Measurement of serum lipids and lipoproteins

Blood was collected into EDTA-vacuutainer tubes and centrifuged at 1560 × *g* for 10 min at 22°C to obtain plasma and blood leukocytes. To obtain serum for lipid measurements, blood was collected into SST-vacuutainer tubes, allowed to clot at room temperature for 20 min and centrifuged at 1560 × *g* for 10 min at 22°C. Total cholesterol (TC) and triglycerides (TG) were measured using enzymatic methods on an Ortho Clinical Diagnostics Vitros 250. High density lipoprotein cholesterol (HDLC) was measured using a direct enzymatic method (Beckman Coulter) on the Synchron LX20PRO analyzer (Beckman Coulter) and LDLC was calculated by the Friedewald equation.

### Genotyping

Genomic DNA was isolated from blood leukocytes using QIAamp DNA Blood Kit (Qiagen Sciences, MD). Primer sequences and polymerase chain reaction (PCR) for amplification of the individual exons of the *PCSK9* gene were as per Abifadel *et al.* (2003). Standard DNA-sequencing reactions were carried out as a service by BioBasic Sequencing (Markham, On, CAN).

### Measurement of serum PCSK9

The serum PCSK9 assay was carried out using a human PCSK9 ELISA from CyClex Co (Japan). All samples were quantified 4× with an intra-assay coefficient of variability (CV) of 1.5-2.6% and an interassay CV of 2.9-7.1%.

### Statistical analyses

Results are expressed as means ± SD except where indicated. Spearman correlation coefficients (*r*) were determined to assess the relationship between different parameters. The unpaired Student *t*-test, Mann Whitney test or Kruskal Walis test (with Dunn’s multiple comparison post test) was used for statistical analyses of differences as appropriate and indicated. Minor allele frequencies were compared using Fisher’s Exact and Chi-squared tests. Data were analysed using Graphpad Prism 5 software (La Jolla, CA) and significance defined as p < 0.05.

## Abbreviations

PCSK9: Proprotein convertase subtilisin/kexin 9; LDLC: Low density lipoprotein cholesterol; LDLR: Low density lipoprotein receptor; APOB100: Apolipoprotein B100; ADH: Autosomal dominant hypercholesterolemia; MAF: Minor allele frequency; FH: Familial hypercholesterolemia; BMI: Body mass index; TC: Total cholesterol; TG: Triglycerides; HDLC: High density lipoprotein cholesterol; PCR: Polymerase chain reaction; ELISA: Enzyme linked immunosorbant assay; SD: Standard deviation

## Competing interests

The authors declare they have no competing interests. JD is employed by or has a leadership role in Quebec Consortium on Drug Discovery (CQDM), Residual Risk Reduction Initiative Foundation (R3i), consulting or advisory role with Abbott/Solvay, AstraZeneca, Amgen, Acasti, Amgen, Cortria, Genzyme, McCain, Merck, Pfizer, and Roche and has received honoraria from Abbott/Solvay, AstraZeneca-BMS Alliance, Acasti, Amgen, McCain, Merck, Pfizer, and Roche. Funding organizations did not play any role in study design, in the collection, analyses, or interpretation of data, or in the writing and submission of the report for publication.

## Authors’ contributions

JM collected, analysed and compiled study data, had full access to all data in the study, and had final responsibility for preparation and submission of this manuscript for publication, TCO supervised the Ottawa recruitment, lipoprotein studies, compilation of lipoprotein data and participated in preparation of the manuscript, AR carried out PCSK9 genotyping and PCSK9 ELISA and participated in preparation of the manuscript, MC^b^ recruited subjects for the Ottawa recruitment, compiled the lipoprotein data and contributed to the manuscript review, LB participated in the recruitment, collection and analysis of the PCSK9 human data from Montreal and manuscript review, TD carried out PCSK9 genotyping and PCSK9 ELISA and manuscript review, FS participated in the collection and analysis of the PCSK9 human data and manuscript review, MM participated in the recruitment, collection and analysis of the PCSK9 human data and manuscript review, JD supervised the Montreal recruitment, lipoprotein studies, compilation of lipoprotein data and manuscript review, MC^ac^ co-supervised Ottawa and Montreal recruitment, participated in data compilation and contributed to the manuscript preparation. All authors read and approved the final manuscript.

## Supplementary Material

Additional file 1: Figure S1 The relationship between serum PCSK9 and lipoprotein parameters, age and body mass index (BMI) by Spearmen correlation (*r*) and significance (*p*) using GraphPad Prism 5 Software. Click here for file
