# Supplementary material for: Differential effects of PCSK9 loss of function variants on serum lipid and PCSK9 levels in Caucasian and African Canadian populations
Source: Lipids Health Dis. 2013 May 10;12:70. doi: 10.1186/1476-511X-12-70 (PMC3661383; doi:10.1186/1476-511X-12-70)
Supplement: Additional file 1: Figure S1 — The relationship between serum PCSK9 and lipoprotein parameters, age and body mass index (BMI) by Spearmen correlation (r) and significance (p) using GraphPad Prism 5 Software. [file 1476-511X-12-70-S1.ppt]

## Slide 1
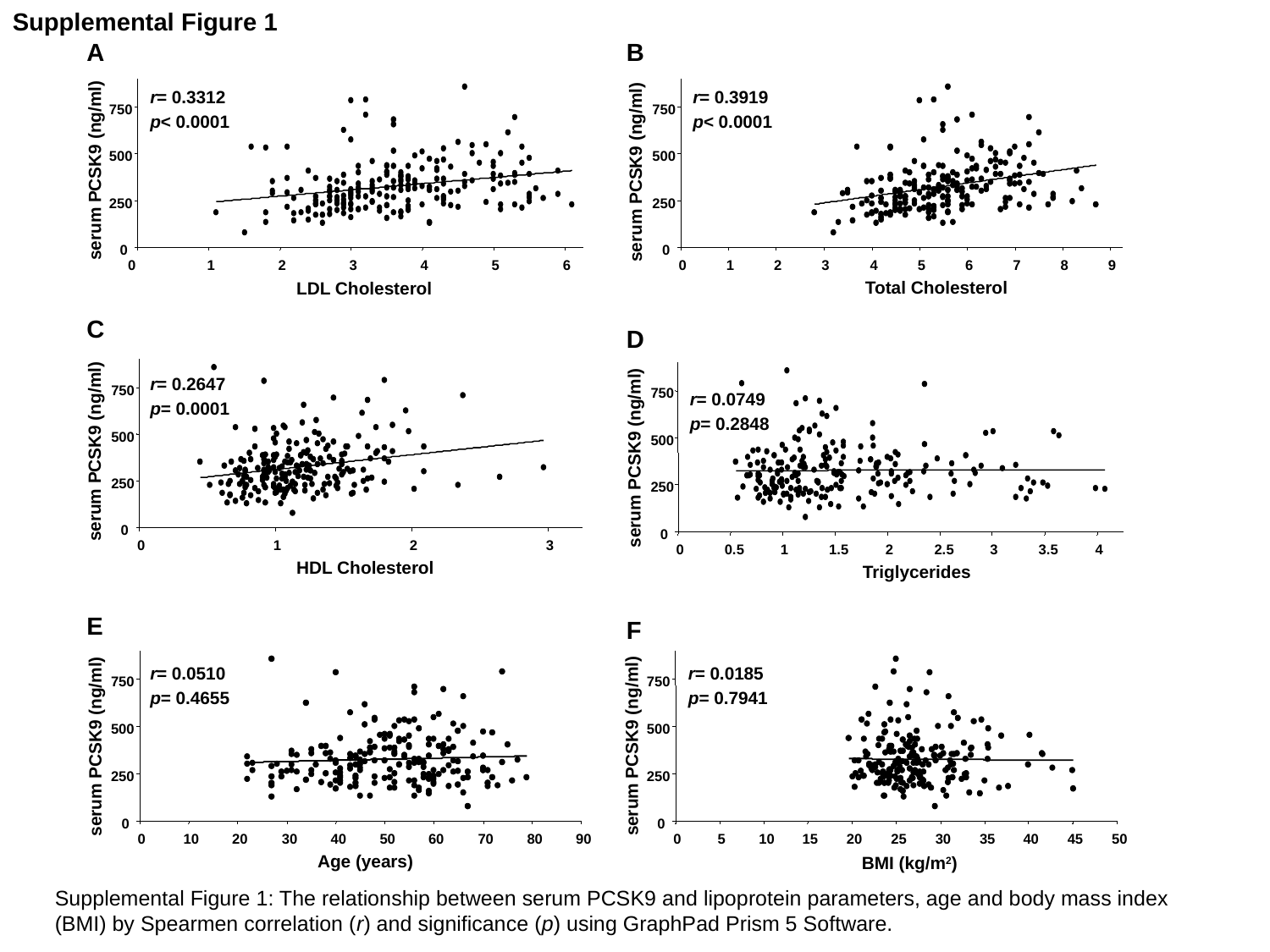

Supplemental Figure 1
A
750
500
250
0
0
1
2
3
4
5
6
serum PCSK9 (ng/ml)
LDL Cholesterol
B
750
500
250
0
0
1
2
3
4
5
6
7
8
9
serum PCSK9 (ng/ml)
 Total Cholesterol
r= 0.3312
p< 0.0001
r= 0.3919
p< 0.0001
C
750
500
serum PCSK9 (ng/ml)
250
0
0
1
2
3
HDL Cholesterol
750
500
250
0
0
0.5
1
1.5
2
2.5
3
3.5
4
serum PCSK9 (ng/ml)
Triglycerides
D
r= 0.2647
p= 0.0001
r= 0.0749
p= 0.2848
750
500
250
0
0
10
20
30
40
50
60
70
80
90
E
serum PCSK9 (ng/ml)
Age (years)
750
500
250
0
0
5
10
15
20
25
30
35
40
45
50
serum PCSK9 (ng/ml)
BMI (kg/m2)
F
r= 0.0510
p= 0.4655
r= 0.0185
p= 0.7941
Supplemental Figure 1: The relationship between serum PCSK9 and lipoprotein parameters, age and body mass index (BMI) by Spearmen correlation (r) and significance (p) using GraphPad Prism 5 Software.
